# Supplementary material for: Healthcare workers’ views on mandatory SARS-CoV-2 vaccination in the UK: A cross-sectional, mixed-methods analysis from the UK-REACH study
Source: eClinicalMedicine. 2022 Mar 15;46:101346. doi: 10.1016/j.eclinm.2022.101346 (PMC8923694; doi:10.1016/j.eclinm.2022.101346)
Supplement: Supplementary file 1 [file mmc1.pdf]

Supplementary Information

Healthcare workers' views on mandatory SARS-CoV-2 vaccination in the United Kingdom: a cross-sectional, mixed-methods analysis from the UK-REACH study

Katherine Woolf[1], Mayuri Gogoi[2], Christopher A Martin[2,3], Padmasayee Papineni[4], Susie Lagrata[5], Laura B Nellums[6], I Chris McManus[1], Anna L Guyatt[7], Carl Melbourne[8], Luke Bryant[2], Amit Gupta[9], Catherine John[7], Sue Carr[10 11], Martin D Tobin[7], Sandra Simpson[12], Bindu Gregary[13], Avinash Aujayeb[14], Stephen Zingwe[15], Rubina Reza[16], Laura J Gray[7], Kamlesh Khunti[17], Manish Pareek[2,3]*.

On behalf of the UK-REACH Study Collaborative Group+

*Corresponding author manish.pareek@leicester.ac.uk

[1]Research Department of Medical Education, University College London Medical School

[2]Department of Respiratory Sciences, University of Leicester, Leicester, UK

[3]Department of Infection and HIV Medicine, University Hospitals of Leicester NHS Trust, Leicester, UK

[4]Ealing Hospital, London North West University Healthcare NHS Trust, Southall, UK

[5]University College London Hospitals NHS Foundation Trust, London, UK

[6] Division of Epidemiology and Public Health, School of Medicine, University of Nottingham.

[7]Department of Health Sciences, University of Leicester, Leicester, UK

[8]Genetic Epidemiology Research Group, Department of Health Sciences, University of Leicester, Leicester, UK

[9]Oxford University Hospitals NHS Foundation Trust.

[10]University Hospitals Leicester NHS Trust, Leicester Royal Infirmary.

[11]General Medical Council.

[12]Nottinghamshire Healthcare NHS Foundation Trust.

[13] Lancashire Clinical Research Facility, Royal Preston Hospital.

[14]Respiratory department, Northumbria Specialist Emergency Care Hospital.

[15]Research and Development Department, Berkshire Healthcare NHS Foundation Trust.

[16]Derbyshire Healthcare NHS Foundation Trust Centre for Research and Development, Kingsway Hospital site.

[17]Diabetes Research Centre, University of Leicester.

+Manish Pareek (Chief investigator), Laura Gray (University of Leicester), Laura Nellums (University of Nottingham), Anna L Guyatt (University of Leicester), Catherine John (University of Leicester), I Chris McManus (University College London), Katherine Woolf (University College London), Ibrahim Akubakar (University College London), Amit Gupta (Oxford University Hospitals), Keith R Abrams (University of Warwick), Martin D Tobin (University of Leicester), Louise Wain (University of Leicester), Sue Carr (University Hospital Leicester), Edward Dove (University of Edinburgh), Kamlesh Khunti (University of Leicester), David Ford (University of Swansea), Robert Free (University of Leicester).

Supplementary Table 1. Description of the cohort stratified by coded response to the free text question and selected predictors

Variable	Total	Code							P value†
		Do nothing	Educate, increase access or incentivise	Maintain restrictions	Specific restrictions for unvaccinated	Mandatory vaccination for HCW / social care staff	Mandatory vaccination for general population or limited access to vital services	Don't know	
	**N=3235**	**580 (17.9)**	**1047 (32.4)**	**336 (10.4)**	**547 (16.9)**	**201 (6.2)**	**377 (11.7)**	**147 (4.5)**	
**Age**, med(IQR)	46 (35 – 55)	46 (36 – 56)	48 (38 – 56)	41 (32 – 52)	46 (35 – 56)	54 (46 – 61)	49 (38 – 57.5)	46 (35 – 56)	<0.001
Missing	13 (0.4)	6 (1.0)	2 (0.2)	0 (0.0)	2 (0.4)	2 (1.0)	1 (0.3)	0 (0.0)	
**Sex**									
Male	824 (25.5)	132 (22.8)	292 (27.9)	65 (19.4)	138 (25.2)	43 (21.4)	128 (34.0)	26 (17.7)	<0.001
Female	2405 (74.3)	445 (76.7)	754 (72.0)	271 (80.7)	408 (74.6)	158 (78.6)	248 (65.8)	121 (82.3)	
Missing	6 (0.2)	3 (0.5)	1 (0.1)	0 (0.0)	1 (0.2)	0 (0.0)	1 (0.3)	0 (0.0)	
**Ethnicity**									
White	2336 (72.2)	444 (76.6)	688 (65.7)	231 (68.8)	411 (75.1)	170 (84.6)	275 (72.9)	117 (79.6)	<0.001
Asian	571 (17.7)	78 (13.5)	236 (22.5)	71 (21.1)	84 (15.4)	16 (8.0)	70 (18.6)	16 (10.9)	
Black	122 (3.8)	28 (4.8)	52 (5.0)	10 (3.0)	16 (2.9)	3 (1.5)	9 (2.4)	4 (2.7)	
Mixed	144 (4.5)	26 (4.5)	47 (4.5)	15 (4.5)	27 (4.9)	7 (3.5)	16 (4.2)	6 (4.1)	
Other	60 (1.9)	3 (0.5)	24 (2.3)	9 (2.7)	9 (1.7)	5 (2.5)	7 (1.9)	3 (2.0)	
Missing	2 (0.1)	1 (0.2)	0 (0.0)	0 (0.0)	0 (0.0)	0 (0.0)	0 (0.0)	1 (0.7)	
**Migration status**									
Born in the UK	2356 (72.8)	447 (77.1)	709 (67.7)	233 (69.4)	424 (77.5)	164 (81.6)	267 (70.8)	112 (76.2)	<0.001
Born outside the UK	791 (24.5)	120 (20.7)	306 (29.2)	91 (27.1)	114 (20.8)	33 (16.4)	101 (26.8)	26 (17.7)	
Missing	88 (2.7)	13 (2.2)	32 (3.1)	12 (3.6)	9 (1.7)	4 (2.0)	9 (2.4)	9 (6.1)	
**IMD quintile**									
1 (most deprived)	275 (8.5)	55 (9.5)	88 (8.4)	42 (12.5)	33 (6.0)	9 (4.5)	36 (9.6)	12 (8.2)	0.002
2	472 (14.6)	102 (17.6)	147 (14.0)	53 (15.8)	60 (11.0)	29 (14.4)	55 (14.6)	26 (17.7)	
3	588 (18.2)	99 (17.1)	193 (18.4)	59 (17.6)	96 (17.6)	36 (17.9)	78 (20.7)	27 (18.4)	
4	727 (22.5)	117 (20.2)	243 (23.2)	78 (23.2)	136 (24.9)	36 (17.9)	84 (22.3)	33 (22.5)	
5 (least deprived)	808 (25.0)	124 (21.4)	283 (27.0)	67 (19.9)	149 (27.2)	68 (33.8)	86 (22.8)	31 (21.1)	

Missing	365 (11.3)	83 (14.3)	93 (8.9)	37 (11.0)	73 (13.4)	23 (11.4)	38 (10.1)	18 (12.2)	
**Job role**									
Medical	773 (23.9)	95 (16.4)	301 (28.8)	63 (18.8)	133 (24.3)	55 (27.4)	99 (26.3)	27 (18.4)	<0.001
Nursing (inc Midwives + HCAs)	698 (21.6)	139 (24.0)	196 (18.7)	75 (22.3)	115 (21.0)	52 (25.9)	82 (21.8)	39 (26.5)	
AHPs*	1303 (40.3)	247 (42.6)	416 (39.7)	155 (46.1)	218 (39.9)	65 (32.3)	135 (35.8)	67 (45.6)	
Dental	173 (5.4)	38 (6.6)	45 (4.3)	21 (6.3)	27 (4.9)	14 (7.0)	22 (5.8)	6 (4.1)	
Administrative/estates/other	195 (6.0)	42 (7.2)	58 (5.5)	16 (4.8)	39 (7.1)	10 (5.0)	24 (6.4)	6 (4.1)	
Missing	93 (2.9)	19 (3.3)	31 (3.0)	6 (1.8)	15 (2.7)	5 (2.4)	15 (4.0)	2 (1.4)	
**Exposure to patients with COVID-19 (at time of second questionnaire)**									
No contact/remote contact only	2518 (77.8)	458 (79.0)	809 (77.3)	249 (74.1)	431 (78.8)	165 (82.1)	291 (77.2)	115 (78.2)	0.26
Face to face but no physical contact	149 (4.6)	29 (5.0)	59 (5.6)	19 (5.7)	23 (4.2)	6 (3.0)	11 (2.9)	2 (1.4)	
Physical contact	377 (11.7)	68 (11.7)	116 (11.1)	43 (12.8)	65 (11.9)	16 (8.0)	49 (13.0)	20 (13.6)	
missing	191 (5.9)	25 (4.3)	63 (6.0)	25 (7.4)	28 (5.1)	14 (7.0)	26 (6.9)	10 (6.8)	
**SARS-CoV-2 vaccine hesitancy**									
Not hesitant	2239 (69.2)	333 (57.4)	750 (71.6)	206 (61.3)	412 (75.3)	174 (86.6)	285 (75.6)	79 (53.7)	<0.001
Hesitant	862 (26.7)	236 (40.7)	244 (23.3)	112 (33.3)	116 (21.2)	20 (10.0)	78 (20.7)	56 (38.1)	
Missing	134 (4.1)	11 (1.9)	53 (5.1)	18 (5.4)	19 (3.5)	7 (3.5)	14 (3.7)	12 (8.2)	
**Number of influenza vaccines in previous 2 seasons**									
0	456 (14.1)	119 (20.5)	142 (13.6)	48 (14.3)	64 (11.7)	16 (8.0)	43 (11.4)	24 (16.3)	<0.001
1	515 (15.9)	100 (17.2)	174 (16.6)	54 (16.1)	84 (15.4)	30 (14.9)	57 (15.1)	16 (10.9)	
2	2123 (65.6)	342 (59.0)	682 (65.1)	218 (64.9)	382 (69.8)	147 (73.1)	259 (68.7)	93 (63.3)	
Missing	141 (4.4)	19 (3.3)	49 (4.7)	16 (4.8)	17 (3.1)	8 (4.0)	18 (4.8)	18 (4.8)	
**VAX score,** med (IQR)	16 (14 – 17)	15 (13 – 17)	16 (14 – 17)	15 (13 – 17)	16 (15 – 17)	16 (15 – 18)	16 (14 – 18)	16 (13 – 17)	<0.001
Missing	0 (0.0)	0 (0.0)	0 (0.0)	0 (0.0)	0 (0.0)	0 (0.0)	0 (0.0)	0 (0.0)	
**Number of comorbidities**									
0	2465 (76.2)	451 (77.8)	810 (77.4)	258 (76.8)	412 (75.3)	147 (73.1)	285 (75.6)	102 (69.4)	0.9
≥1	249 (7.7)	43 (7.4)	78 (7.5)	27 (8.0)	41 (7.5)	20 (10.0)	28 (7.4)	12 (8.2)	
Missing	521 (16.1)	86 (14.8)	159 (15.2)	51 (15.2)	94 (17.2)	34 (16.9)	64 (17.0)	33 (22.5)	
**Pregnant**[‡]									
Not pregnant	2947 (91.1)	539 (92.9)	938 (89.6)	301 (89.6)	505 (92.3)	187 (93.0)	350 (92.8)	127 (86.4)	0.16

Pregnant	63 (2.0)	12 (2.1)	20 (1.9)	12 (3.6)	12 (2.2)	3 (1.5)	2 (0.5)	2 (1.4)	
Missing	225 (7.0)	29 (5.0)	89 (8.5)	23 (6.9)	30 (5.5)	11 (5.5)	25 (6.6)	18 (12.2)	
**Previous COVID-19**									
Never tested	278 (8.6)	55 (9.5)	99 (9.5)	31 (9.2)	37 (6.8)	21 (10.5)	28 (7.4)	7 (4.8)	0.22
Tested negative	2181 (67.4)	380 (65.5)	708 (67.6)	238 (70.8)	376 (68.7)	127 (63.2)	256 (67.9)	96 (65.3)	
Tested positive	763 (23.6)	141 (24.3)	237 (22.6)	65 (19.4)	133 (24.3)	51 (25.4)	92 (24.4)	44 (29.9)	
Missing	13 (0.4)	4 (0.7)	3 (0.3)	2 (0.6)	1 (0.2)	2 (1.0)	1 (0.3)	0 (0.0)	
**Lives with a person ≥65 years old**									
No	2808 (86.8)	510 (87.9)	902 (86.2)	294 (87.5)	483 (88.3)	166 (82.6)	328 (87.0)	125 (85.0)	0.25
Yes	320 (9.9)	56 (9.7)	108 (10.3)	29 (8.6)	52 (9.5)	30 (14.9)	35 (9.3)	10 (6.8)	
Missing	107 (3.3)	14 (2.4)	37 (3.5)	13 (3.9)	12 (2.2)	5 (2.5)	14 (3.7)	12 (8.2)	
**Lives with other key workers**									
No	1678 (51.9)	287 (49.5)	560 (53.5)	168 (50.0)	283 (51.7)	118 (58.7)	182 (48.3)	80 (54.4)	0.05
Yes	1444 (44.6)	279 (48.1)	450 (43.0)	155 (46.1)	250 (45.7)	74 (36.8)	183 (48.5)	53 (36.1)	
Missing	113 (3.5)	14 (2.4)	37 (3.5)	13 (3.9)	14 (2.6)	9 (4.5)	12 (3.2)	14 (9.5)	
**Trusts employing organisation to deal with concern about unsafe clinical practice**									
Does not trust organisation	844 (26.1)	165 (28.5)	248 (23.7)	88 (26.2)	149 (27.2)	45 (22.4)	116 (30.8)	33 (22.5)	0.07
Trusts organisation	2161 (66.8)	384 (66.2)	724 (69.2)	223 (66.4)	361 (66.0)	138 (68.7)	230 (61.0)	101 (68.7)	
Missing	230 (7.1)	31 (5.3)	75 (7.2)	25 (7.4)	37 (6.8)	18 (9.0)	31 (8.2)	13 (8.8)	
**COVID-19 conspiracies score,** med (IQR)	8 (7 – 10)	9 (8 – 11)	8 (7 – 10)	9 (8 – 10)	8 (7 – 10)	8 (7 – 9)	9 (7 – 10)	9 (8 – 10)	<0.001
Missing	128 (4.0)	17 (2.9)	44 (4.2)	16 (4.8)	16 (2.9)	6 (3.0)	15 (4.0)	14 (9.5)	

Also includes pharmacists, healthcare scientists, ambulance workers and those in optical roles. † groups compared using chi-squared tests for categorical variables and Kruskal-Wallis tests for continuous variables. Values expressed as n(%) unless stated otherwise. ‡ Participants are coded as pregnant if they indicated they were pregnant at baseline or at follow up (i.e. this category includes participants who were pregnant at baseline and who may have delivered by the time of completing the follow up questionnaire. IMD – index of multiple deprivation; IQR – interquartile range;

Supplementary Table 2. Comparing demographic and occupational characteristics of free text responders and non-responders

	Total N=5633	Responders N=3235	Non-responders N=2398	P value*
**Age, med(IQR)**	46 (35 – 55)	47 (36 – 56)	43 (34 – 53)	<0.001
**Sex**				
Male	1411 (25.1)	824 (25.5)	587 (24.5)	0.38
Female	4215 (74.8)	2405 (74.3)	1810 (75.5)	
Missing	7 (0.1)	6 (0.2)	1 (0.0)	
**Ethnicity**				
White	4,106 (72.9)	2336 (72.2)	1770 (73.8)	0.32
Asian	984 (17.5)	571 (17.7)	413 (17.2)	
Black	197 (3.5)	122 (3.8)	75 (3.1)	
Mixed	236 (4.2)	144 (4.5)	92 (3.8)	
Other	103 (1.8)	60 (1.9)	43 (1.8)	
Missing	7 (0.1)	2 (0.1)	5 (0.2)	
**Job role**				
Medical	1365 (24.2)	778 (24.1)	587 (24.5)	0.22
Nursing (inc Midwives + HCAs)	1160 (20.6)	698 (21.6)	462 (19.3)	
AHPs	2277 (40.4)	1301 (40.2)	976 (40.7)	
Dental	325 (5.8)	171 (5.3)	154 (6.4)	
Administrative/estates/other	321 (5.7)	184 (5.7)	137 (5.7)	
Missing	185 (3.3)	103 (3.2)	82 (3.4)	

* Responders and non-responders compared using chi-squared tests for categorical variables and Wilcoxon rank-sum tests for continuous variables. Values expressed as n(%) unless stated otherwise.
